# CSRR Structure Design for NV Spin Manipulation with Microwave Strength and Fluorescence Collection Synchronous Enhancement

**DOI:** 10.3390/ma16103718

**Published:** 2023-05-14

**Authors:** Yanjie Gao, Hao Guo, Huanfei Wen, Zhonghao Li, Zongmin Ma, Jun Tang, Jun Liu

**Affiliations:** State Key Laboratory of Dynamic Measurement Technology, Shanxi Province Key Laboratory of Quantum Sensing and Precision Measurement, North University of China, Taiyuan 030051, China

**Keywords:** complementary split ring resonator, strong uniform microwave manipulation, nitrogen vacancy, fluorescence collection efficiency improvement

## Abstract

In this work, we designed, simulated, and tested a complementary split ring resonator (CSRR) for the purpose of applying a strong and uniform microwave field for the manipulation of nitrogen vacancy (NV) ensembles. This structure was fabricated by etching two concentric rings on a flat metal film that was deposited on a printed circuit board. A metal transmission on the back plane was used as the feed line. The fluorescence collection efficiency was improved by about 2.5 times with the CSRR structure compared to that without CSRR. Furthermore, the maximum Rabi frequency could reach 11.3 MHz, and the Rabi frequency variation was smaller than 2.8% in an area of 250 × 75 μm. This could pave the way to achieving high-efficiency control of the quantum state for spin-based sensor applications.

## 1. Introduction

A nitrogen vacancy is a point defect with C3V symmetry in a diamond matrix with excellent physical sensitivity and stability for quantum metrology, communication, and computation [1,2]. In these scenarios, a microwave field is an important component to manipulate the quantum state of the NV into a specific state [3,4,5]. Microwaves provide the oscillating magnetic field to rotate the axis of the quantum state, as designed in a Bloch sphere. Compared with all-optical and electrical methods, which have the limitations of only being applied in a single NV center or being hard to employ for complex quantum state manipulation [6,7,8], the microwave method provides better precision in quantum state rotation. Quantum metrology with an NV combining quantum state manipulation through microwaves has achieved excellent sensitivity in the areas of nuclear magnetic resonance (NMR), as well as magnetic and electric sensing [9,10,11,12,13,14,15,16].

In a single-NV application, a metal wire deposited onto a diamond near the NV defect is enough to release the microwave field without consideration of uniformity. However, an NV ensemble with N NVs can provide better sensitivity, down to 1/√N [17]. Uniformity and strength of the microwave should be properly weighed in ensemble applications.

In addition to the precise control of quantum states, fluorescence collection efficiency is also vital to quantum metrology with NVs. Although the chemical inertness of diamond provides a good protection for this quantum sensor, the refractive index of 2.4 in diamond limits the extraction efficiency of fluorescent photons. Both in sensing applications with a single color center or ensemble color centers, different technologies are used to increase the number of photons collected to improve sensitivity. The solid immersion lens structure is often used in the sensing system with single-color center to improve the fluorescence collection efficiency, and the counting rate could reach 4 MHz [18]; at the same time, there are also methods to increase the photon emission rate by using metal antennas, where the simulation results show that the counting rate can be increased by three orders [19].

However, these enhancement methods in a single-color center are difficult to apply in ensemble applications. In ensemble applications, the fluorescence could be enhanced by increasing the optical path through total internal reflection after the diamond is chamfered [20], coating gold film on the back of the diamond to reflect fluorescence [21], or adding prisms on the four sides of the diamond to increase the fluorescence collection effect [22]. In these efforts of improving collection efficiency of fluorescence, complex components or expensive processes are introduced. The structure designed and prepared for the ensemble sensing application in this paper has properties of robustness, easy integration, and achievement of similar efficiency improvement to other methods.

After summarizing and analyzing previous work, we designed a complementary split ring resonator for a strong uniform microwave delivery and enhanced fluorescence collection efficiency. In the experiment, the Rabi frequency could reach 11.3 MHz with good homogeneity; its deviation in 250 × 75 μm did not exceed 2.8%. With the reflection of the metal layer, the collection efficiency could be improved by 2.5 times compared to that before in the process of measuring the fluorescence spectrum. The improved fluorescence collection efficiency could promote better sensitivity, and the uniform strong microwave field provided can avoid decoherence when preparing the ensemble NVs as a certain quantum state. Therefore, the microwave field with strong uniform radiation and better fluorescence collection efficiency can promote the use of complex pulse sequences in the relevant sensing scenario. The research results of this paper can be applied to many sensor applications with NV ensembles, such as the measurement of temperature, magnetic field, electric field, angular rate, etc.

## 2. Nitrogen Vacancy and Complementary Split Ring Resonator

**Figure 1:**
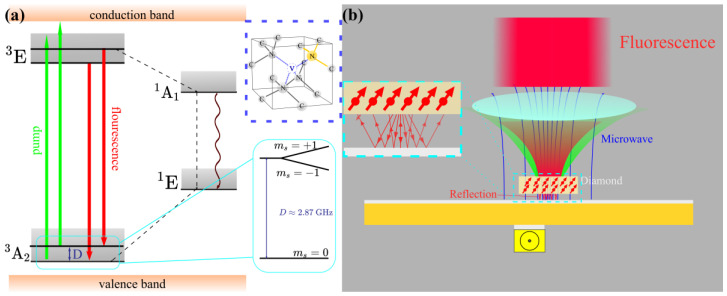
(**a**) Fundamental physical property of NV. (**b**) Microwave irradiation and fluorescence reflection of CSRR.

The fundamental physical property of an NV is illustrated by Figure 1a. An NV in a ground state could be pumped to transition to an excited state and radiate fluorescence outward in the process of the excited state returning to the ground state. The fluorescence intensity is related to quantum state, because NVs of different quantum states have different paths from the excited state to the ground state. The energy level splitting in the ground state is related to the magnetic field. The picture in the blue box is the structure of the NV color center in the diamond lattice, with four orientations. A brief schematic of the CSRR applied in the measurement with the NV is shown in Figure 1b. Diamond is placed in the dense microwave irradiation region. Fluorescence that is transmitted away from the objective lens is reflected to the location of the objective.

**Figure 2:**
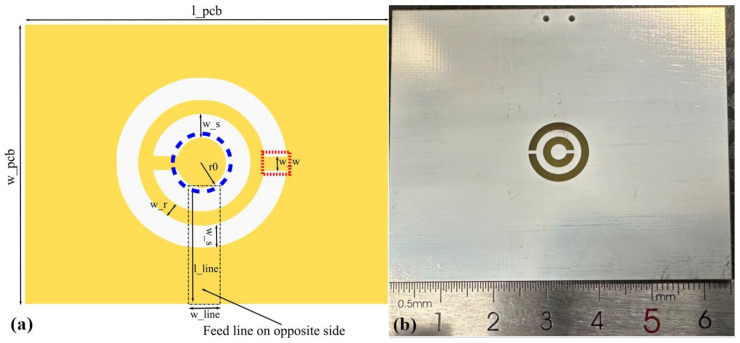
(**a**) Schematic diagram of CSRR. (**b**) Photograph of CSRR.

A CSRR is one of the planar metamaterials that can respond to a microwave field within a certain frequency [23,24,25,26], and it was first proposed as a complementary screen for a split ring resonator (SRR) [27,28]. In the design of a CSRR, two concentric rings are etched from a metal sheet. In the analysis of a CSRR, researchers can switch the inductance and capacitance in the equations of an SRR with the same dimensions to obtain the relevant parameter results. When a CSRR is loaded behind a transmission line, the metamaterial will respond to the specific alternative electromagnetic field according to the relative angle between the symmetry plane of the CSRR and the transmission line. Based on this consideration, a metal rectangular patch for connecting the ring and circle is perpendicular to the transmission line, as shown in Figure 2a. The fabricated resonator is shown in Figure 2b. CSRR was fabricated through a printed circuit board process. Copper was deposited on the FR4 substrate to form a CSRR according to the given shape design parameters, and then tin was plated to avoid oxidation of the copper layer.

The initial design for a CSRR was based on the dimensions of the diamond and then refined with electromagnetic analysis software. In the initial idea, a diamond sample was placed on the central circular metal plate, at the position of the blue circle in Figure 2a. Later in the test, the diamond sample was placed above the connection sheet, at the position of the red rectangle in Figure 2a. In the case of approximate central symmetry, the center of the CSRR can provide maximum uniformity for the microwave. In a previous NV gyroscope study, the electron spin resonance point was set to 2.84 GHz [29]. Therefore, in our work, the optimization goal was 2.84 GHz. During the simulation, parameters such as the radius of the central disk, r0, the width of the ring, w_r, the radial distance between metal materials, w_s, the width of the rectangular sheet that joins the metal together, the distance between the feed line endpoints, w_w, and the length of the feed line, l_line, could be adjusted. The substrate material in the simulation and fabrication was FR4, whose dielectric constant was 4.3 and whose thickness was 1.6 mm. The geometrical parameters after optimization in simulation were shown in Table 1.

## 3. Results and Discussion

### 3.1. Simulation and Preliminary Characterization

**Figure 3:**
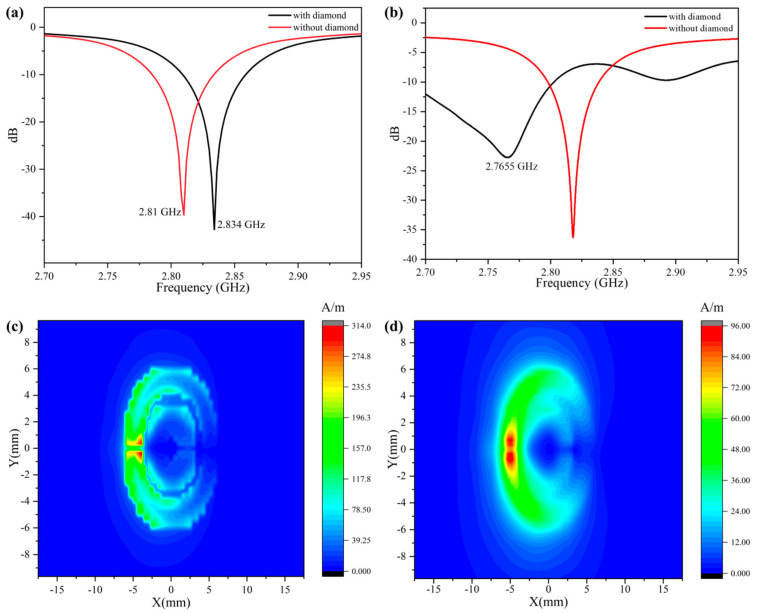
Antenna Performance Schematic. (**a**) S11 parameters simulated with and without diamond on CSRR. (**b**) S11 parameters tested with and without diamond on CSRR. (**c**) Diagram of magnetic field distribution at 10 μm height. (**d**) Diagram of magnetic field distribution at 500 μm height.

The S11 parameter is the return loss of the CSRR, and in the simulation, the lower it was, the better the result was. The bandwidth could not be too narrow to ensure a relatively low quality factor and correspondingly higher response time. In the simulation, the S11 could be −43 dB, and the resonant frequency was 2.834 GHz. When simulating the S11 of the CSRR with a diamond loaded, the S11 with a diamond above the circular metal plate and above the connection sheet was simulated. With the effective dielectric constant of the resonator changed with a diamond, the resonant frequency shifted from 2.834 GHz to 2.81 GHz in both conditions (Figure 3a). The actual test results are shown in Figure 3b. The resonant frequency with no diamond loaded had only a slight shift, which was probably due to minor errors in manufacturing. The actual S11 at the resonant frequency could be −33 dB. However, the curve was severely deformed when the diamond was loaded, as shown in Figure 3b.

With a diamond attached to the antenna, the resonant frequency was shifted greatly and the S11 value was not as good as that in the test results without a diamond. We inferred that the reason for this was that the glue and diamond worked together, which changed the dielectric constant of the surface area of the resonator. The Q of this resonator was 27 (BW~100 MHz). In the application of the microwave manipulation for the NV, a shorter time was required to reach the resonant state, where τ = Q/2 × π × f ~ 1.55 ns, which was optimal for the electron paramagnetic resonance (ESR) and optically detected magnetic resonance (ODMR).

The microwave field intensity would drop with an increasing distance from the top side of the antenna. In the simulation, we took two conditions into consideration with the height of the diamond at 10 μm and 500 μm. The data export step after simulation was set to 0.1 mm. The results in Figure 3c,d are the simulation results corresponding to the field intensity distribution at 10 μm and 500 μm from the CSRR surface. The two sets of simulation data showed consistent field distribution trends, but as the distance increased, the total field strength decreased. The field spread in Figure 3c clearly shows the morphology of the CSRR structure. With the excitation of the feed line on the opposite side of the CSRR, the current was mainly transmitted between the ring and the outermost metal layer. Additionally, only a slight amount of current distribution was seen on the center disk, shown as the blue circle in Figure 2a. As the upper surface of the 10 μm-thick diamond was closer to the CSRR surface, the two-dimensional distribution of the current showed a more obvious particle sense. The 1 W input of the microwave in the simulation could produce a 314 A/m field strength. However, when the distance between the monitor plane and the metal surface in the solver was increased to 500 μm, the distribution of the electromagnetic field became smoother (Figure 3d). This could be explained that when the monitor plane was raised, the vector strength of a point on the monitor plane corresponded to the vector sum of the radiation fields of more point sources on the metal layer. This made the distribution shown in Figure 3d on the plane smoother than that shown in Figure 3c.

### 3.2. Characterization of Fluorescence Collection Efficiency Improvement

After completing the preliminary test, we applied the fabricated CSRR to an ODMR platform for further measurement. We first demonstrated fluorescence collection efficiency improvement with an antenna with flat metal that was placed behind the diamond. In the normal confocal equipment for an NV measurement system, color centers are polarized by a focused 532 nm laser that passes through an objective and is limited to a small volume. The fluorescence emitted is scattered into a 4π solid angle, and only a small portion is collected by the objective placed on one side of the diamond. It should also be noted that diamond has a high reflective index of 2.4, and the resulting total internal reflection angle reduces the fluorescence collection efficiency to an extremely low level. Previous work has been carried out to improve the collection efficiency by coating one side of the diamond with gold film with a thickness of several micrometers without affecting the microwave irradiation strength [30]. The antenna in this work could be another way to improve the collection efficiency with an easier-to-implement manufacturing process.

**Figure 4:**
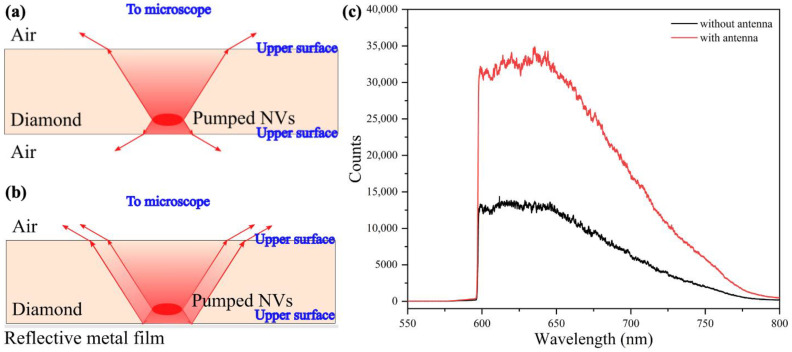
Schematic diagram of the fluorescence collection enhancement effect of the CSRR. (**a**) Diagram of Fluorescence Emission of NVs in Diamond without CSRR on the bottom. (**b**) Diagram of Fluorescence Emission of NVs in Diamond with CSRR on the bottom. (**c**) Fluorescence spectra measured with and without CSRR.

In the measurement, a diamond with diameter of 5 mm and thickness of 0.5 mm was used, with an NV^−^ concentration of about 0.3 ppm. As shown in Figure 4a,b, a diamond with two polished sides was placed between an objective and antenna, and the fluorescence from the pumped NVs that was transmitted upward could be collected. When the reflective metal film was placed below the pumped NVs, the fluorescence that was transmitted downward was also reflected upward and the signal light flux collected by the objective was increased. In this verification experiment, the diamond surface and the antenna metal layer only needed to be smooth, with no defects and scratches, and without additional polishing.

This specific experimental verification was realized with a fluorescence signal acquisition system, which is composed of a 532 nm laser, a microscope, and a fluorescence spectrometer with a refrigerated charge-coupled device (CCD). A 590 nm long pass filter was placed in the system, and fluorescence with wavelength greater than 590 nm could be observed spectrally (Figure 4c). During the spectral acquisition process, the settings of the pump light intensity, focusing position of the objective, and slit setting of the spectrometer remained unchanged. The slit in the spectrometer was set at 10 μm to filter out external stray light, while the only variable was whether there was an antenna below the diamond. The experimental results showed that there could be a fluorescence collection efficiency improvement of approximately 2.5 times and the NV^−^/NV^0^ was low in this sample, as the zero-photon line of NV^−^ was not obvious [31]. This is because NV centers in low-nitrogen diamond are more likely to exist in the form of NV^0^ centers. More than three positions with and without metal film were randomly selected to test the fluorescence spectrum collected by the spectrometer after the diamond was placed above. The results of multiple collection showed that the spectra collected from multiple points with metal film are very close in trend and value, while the spectra obtained from multiple points without metal structure are also very close in trend and value. In total, the collection efficiency could be improved as long as metal film was placed below the pumping point.

(1) $$\eta_{pulsed}=\frac{8}{3\sqrt{3}}\frac{\hbar }{g_{e}\mu_{B}}\frac{1}{C_{pulsed}\sqrt{N}}\frac{\sqrt{t_{I}+T_{2}^{*}+t_{R}}}{T_{2}^{*}}$$

Equation (1) is a typical sensitivity expression of the pulse protocol, where $\sqrt{N}$ represents the photons collected in a measurement. It can be intuitively obtained that when the collected photons doubled, the sensitivity could be improved to 0.7 times the initial value. The increase in the collection efficiency of the fluorescent photons was helpful for the reading of the quantum states in an optical manner.

### 3.3. Characterization of Microwave Strength and Uniformity with CSRR

Then, we tested the strength and variation of microwave with the Rabi sequence. The experimental setup was a normal measurement confocal system for ensemble NVs. All components in the measurement system were commercially available, such as the laser, AOM, filter, photodetector, etc. The data were recorded by an oscillator, and ODMR and Rabi were extracted by data processing on a computer. The diamond sample here was from element six, and the NV concentration was 0.3 ppm.

In the experiment, a diamond sample was first placed on the central disk of the CSRR with expectation that the ODMR spectrum could be measured there. The reason was that we had performed the experiment by measuring the field intensity distribution on the CSRR surface using a microwave probe-linked spectrometer. The output signal of the probe at the center of the CSRR gave an accurate frequency and good intensity for the spectrometer. Unfortunately, there was no ODMR spectrum. Then, the diamond was placed above the junction of the ring and the outermost metal layer for the ODMR test, shown as the red rectangle in Figure 2a.

**Figure 5:**
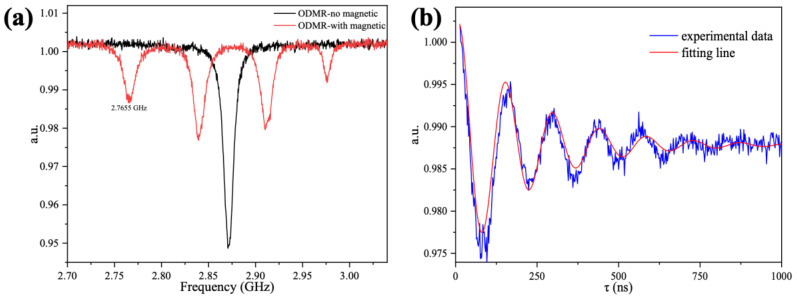
Performance testing of CSRR. (**a**) Optical detection magnetic resonance spectra measured by CSRR. (**b**) Rabi signal measured at 2.7655 GHz with the power in the microwave source set to 0 dBm.

First, the ODMR spectrum was collected with no magnetic field applied. The black line in Figure 5a shows the ODMR without the magnetic field. Under the condition of a strong NV^−^ microwave interaction, no splitting caused by crystal strain in the 2.87 GHz region was observed. Then, the electron energy splitting of the NV^−^ with the external magnetic field was verified by applying a magnetic field with three-axis electromagnetic coils. The direction of the magnetic field was selected as a specific value to maintain a good quantum number for the NV^−^, and the amplitude of the magnetic field was adjusted so that one group of NVs in the diamond had a transition frequency of 2.7665 GHz, as indicated by the red line in Figure 5a.

After the acquisition of the ODMR, the Rabi spectrum was analyzed to obtain the intensity of the specific frequency of the microwave field. The microwave action time for the NV^−^ was swept to acquire the spectrum. The NV^−^ quantum states of |0> and |−1> were taken as a set of quantum bases and mapped in a Bloch sphere. The initial state of the NV^−^ was at |0> after the polarization of the laser. Normally, NV^−^ in the |0> state emits more photons than that in the |−1> state; so, the signal on the Rabi curve oscillated from a high position as the microwave application time increased (Figure 5b). The rapid decay of the signal with the increasing action time could be due to the rapid decoherence caused by the high impurity concentration in the diamond since the natural abundance of 13C and N nuclei that was not converted to NV^−^. The Rabi frequency was determined by nonlinear fitting through exponentially damped sinusoids, as can be seen from the red line in Figure 5b.

**Figure 6:**
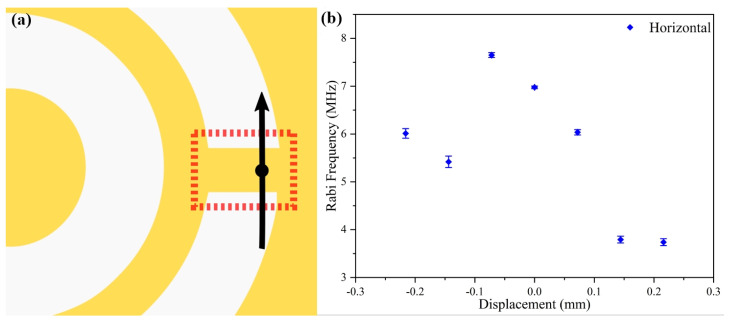
Measurement of distribution of microwave intensity along the horizontal direction. (**a**) Position and direction of measurement. (**b**) Measurement results at 2.7655 GHz.

**Figure 7:**
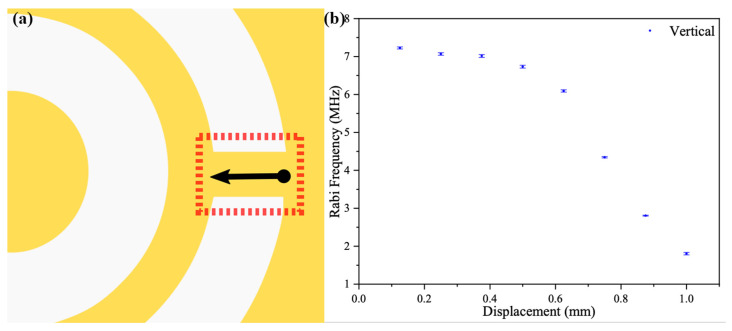
Measurement of distribution of microwave intensity along the vertical direction. (**a**) Position and direction of measurement. (**b**) Measurement results at 2.7655 GHz.

The homogeneity of the radiated MW field of the CSRR in space should be evaluated to provide a reference for applications in quantum sensing with NV. The simulation and preliminary experimental results showed that only the space area above the rectangular metal sheet that connected the metal ring and the outermost metal layer could provide a good ODMR signal, shown as the red rectangle in Figure 2a. We performed the Rabi test along directions parallel to the length and width of the rectangular metal sheet (Figure 6a and Figure 7a). The diamond sample and the CSRR antenna were attached to a three-axis displacement table inside a cramped three-dimensional magnetic coil. After adjusting the distance from the objective to the diamond surface, the z value of the three-axis displacement table was fixed. Then, the x and y values were changed for Rabi acquisition separately to determine the spatial distribution of the MW field.

The amplitude output from a microwave source was fixed at 0 dBm, and the microwave power transmitted to the CSRR had to be within 1–2 W, with consideration of the reflection loss caused by multiple connection nodes in the microwave delivery system and the amplification ability of the microwave amplifier. The width of the rectangular metal sheet connecting the ring and the outermost metal layer was set as 0.4 mm. Then, the 0.4 mm length was split into six approximately equal segments, and the Rabi signal was measured at each node. The starting point was slightly outside the rectangular metal sheet. For previous qualitative experiments, with the assumption of a uniform distribution of NV^−^ in the diamond, the contrast of the ODMR was lower in position away from the metal patch than above the patch. Only the position above the rectangular metal sheet was considered a strong microwave radiation area.

In the case of fixed MW power, laser intensity, and other experimental settings, the Rabi frequency of different nodes above the rectangular metal sheet was recorded, as shown in Figure 6b. The zero point of the coordinate axis was set at the width center of the rectangle, on the black dot in the middle of the black line with arrows, with the positive sides of the rectangle to the right and the negative to the left, as shown in Figure 6a. The Rabi frequency measured at the left edge of the metal sheet was higher than the frequency measured at the right edge. The Rabi frequency was approximately 6 MHz at the start point and approximately 3.7 MHz at the endpoint, and data are shown in Figure 6b. During this measurement, the Rabi frequency tended to move slowly downward in the first two segments, but it tended to be stable in the last part of the right area. In the comparison of the Rabi frequencies for the position of the middle and both sides, the frequency in the middle was about twice as strong as that of both sides. In this situation, the collection of fluorescent photons could be increased while reducing the microwave control time to improve the measurement ability of the quantum system. The two monotonic decreases in the Rabi frequency in the measurement might have been due to the fact that the patch antenna was mounted at an angle to the *y*-axis, causing the final Rabi frequency to appear asymmetrical around the center point. However, the Rabi frequency was almost equal at two points separated by 75 microns on the right side.

In the subsequent test, the focusing point of the objective was moved back to the center of the width of the metal rectangular sheet by adjusting the knob in the y-direction of the three-axis displacement platform. The length of the rectangular metal in the red dotted rectangular box of Figure 7a was 1 mm in the design for the fabricated sample. The length of the metal rectangular sheet was split into seven approximately equal segments, and the Rabi signal was measured at each node identically, as shown in Figure 7. The Rabi frequency measured at the starting point was about 7.2 MHz. The starting point in Figure 7b was defined as the point near the metal ring and the inner circular patch, shown as the black dot at the starting point of the black line with arrows in Figure 7a. The collected signals are shown in Figure 7b. In these measurement results, the collected Rabi frequency first appeared stable in the range from 125 to 375 μm and then decreased monotonically when approaching the outermost metal layer. For the processed one-dimensional data display, compared to the Rabi frequency spreading along the width direction as in Figure 6a, we could not find the Rabi frequency mutation along the length direction. The data in Figure 7b show that the Rabi frequency was able to present a uniform distribution over a range from 125 to 375 μm. Since an assumption was made that the width of the rectangular sheet might not be aligned with the *y*-axis of the three-axis displacement platform, as noted in the previous section, it could also be assumed that the length of the sheet would not be aligned with the *x*-axis of this stage. However, the spreading of the Rabi frequency along the *x*-axis was still smooth. According to Figure 6b and Figure 7b, the Rabi frequency variation was smaller than 2.8% in an area of 250 × 75 μm.

In the currently widely used confocal pumping excitation system, the transverse region of the pumped and excited NV color center is only within a few square microns, which is much smaller than the microwave uniform region of 250 × 75 um in this design. At present, even if a convex lens is used to converge parallel lasers, the laser power density at the focus point is far less than the saturation density of the NV color centers with normal concentration. Therefore, the total number of fluorescence photons collected would not be reduced, and the problem of reducing the total number of signal fluorescence photons will not be encountered temporarily.

Former discussion mainly focused on the microwave distribution in the horizontal plane, and no discussion of microwave distribution along a direction perpendicular to the horizontal plane was conducted. In the confocal optical setup, the size of the longitudinally excited spot is usually more than three times that of the transverse spot. It is difficult to measure the uniformity of the microwave field in the longitudinal direction with the current device. However, the measurement results of uniformity in two-dimensional space in this paper include the longitudinal uniformity. If there is great heterogeneity in the longitudinal direction, the results in the transverse direction are also uneven. In the process of practical use, researchers can prepare diamond films with limited thickness to avoid the limitation of longitudinal microwave field inhomogeneity [32]. These diamond films with NV color centers with limited thickness have been widely investigated and used. Therefore, the lack of microwave longitudinal inhomogeneity analysis in this paper will not limit the use of a complementary split ring resonator in the sensing application of NV color centers.

**Figure 8:**
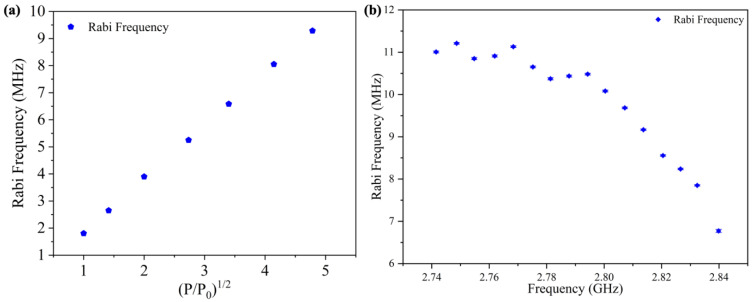
Response Test of CSRR to MW power and frequency. (**a**) Rabi frequency at different microwave input powers, where P is the input power and P_0_ is −12 dBm in microwave source before amplification by the power amplifier module. (**b**) Rabi frequency at different frequencies with fixed input power.

It is certain that the Rabi frequency is proportional to the square root of the MW power. An experiment with a Rabi frequency of 2.7655 GHz for different MW powers was conducted to analyze the performance of the measurement system (Figure 8a). Seven MW powers were chosen as input values to the amplifier, and the corresponding Rabi frequencies were recorded and plotted together. In the condition of reduced MW power, the Rabi amplitude also decreased. The starting point was selected as −12 dBm in order to facilitate the identification and fitting of the Rabi frequency. Then, we changed input power and recorded the Rabi frequency. The data in Figure 8a show that the Rabi frequency increased linearly as the square root of normalized microwave power increased linearly [3,33], (P/P_0_), where P_0_ is −12 dBm at the microwave source output.

To experimentally determine the operating frequency of the CSRR fabricated in this work, a random location above the rectangular sheet was chosen, and the bias magnetic field was adjusted to obtain different resonance frequencies. Due to performance limitations of the electromagnetic system, we could only apply a magnetic field within 4.64 mT. The starting frequency point was 2.84 GHz. At this point, it was possible to clearly distinguish the quarter of the NV that was parallel to the magnetic field from the others. During measurement, the output power of the MW source was set to 6 dBm, the cable connection in the system was fixed, and only the basis magnetic field was adjusted. The measured results are shown in Figure 8b. The Rabi frequency was flat and high from 2.74 GHz to 2.79 GHz and gradually decreased from 2.79 GHz. Even at a frequency of 2.84 GHz, the Rabi frequency could still be 6.5 MHz. This was superior to the performance of the classic Ohm-shaped antenna, whose Rabi frequency was normally around 1.5 MHz. This strong working range from 2.74 GHz to 2.79 GHz also conformed to the design, and it could be applied in the field of the NV sensing. The degenerate energy levels could be lifted in a wide magnetic field, and the quantum measurement with the NV could be more accurate. In addition, since measurement with nanodiamond containing a single NV also requires the application of microwave and collection of fluorescence, a CSRR could also be applied here.

## 4. Conclusions

In conclusion, we designed a CSRR that could provide an improvement in fluorescence collection efficiency with a strong uniform microwave control field. In the spectrum analysis, the fluorescence counts with a CSRR behind the diamond could be improved by 2.5 times compared to those without a CSRR. This could improve the fidelity of the reading quantum state. Additionally, the microwave field irradiated by our CSRR was at least five times stronger than that of a normal omega-shaped microwave irradiation structure. The area of the uniform microwave field being tested was 250 × 75 μm^2^. It is believed that the design and experimental ideas discussed in this paper can provide inspiration for antenna design in the quantum sensing of NVs in diamonds and will be helpful in spin-based sensor applications. The structures with microwave resonance enhancement could be used in conjunction with solid defects with optical ability to read spin states, providing new ideas and references for quantum measurement.

## Figures and Tables

**Table 1:** Geometrical Dimensions of a CSRR with a Designed Resonance Frequency of 2.834 GHz.

Parameter	Value (mm)
ε_r_	4.3
h_pcb	1.6
l_pcb	49.4
w_pcb	65
w_w	0.6
w_line	3.2
l_line	16.3
w_s	1.5
r0	1.5
w_r	1.3

## Data Availability

Not applicable.
